# Enhanced Functions of Peripheral γδ T Cells in Chronic Hepatitis B Infection during Interferon α Treatment *In Vivo* and *In Vitro*

**DOI:** 10.1371/journal.pone.0120086

**Published:** 2015-03-16

**Authors:** Min Chen, Peng Hu, Ning Ling, Hui Peng, Yu Lei, Huaidong Hu, Dazhi Zhang, Hong Ren

**Affiliations:** 1 Institute for Viral Hepatitis, Key Laboratory of Molecular Biology for Infectious Diseases, Ministry of Education, Second Affiliated Hospital, Chongqing Medical University, Chongqing, China; 2 Department of laboratory medicine, Second Affiliated Hospital, Chongqing Medical University, Chongqing, China; Harvard Medical School, UNITED STATES

## Abstract

**Background:**

γδ T cells play an important role in infectious, autoimmune, or neoplastic diseases. Here, a study was conducted to investigate the dynamic changes in phenotype and function of peripheral γδ T cells in patients with chronic hepatitis B (CHB) during pegylated-interferon (pegIFN)-α treatment, and to explore their roles in IFN-α therapy.

**Methods:**

Total 15 CHB patients with pegIFN-α therapy and 6 healthy controls (HC) were enrolled in this study. Flow cytometry was used for the study of frequency of peripheral γδ T cells, subtypes, effector or memory γδ T cells, and also the IFN-γ+, TNF-α+, CD107a+ or Granzyme B+ γδ T cells in 10 patients at week 0, 4, 8, 12, 24, 36 and 48 of treatment. Another 5 CHB patients and 6 HC were recruited for the γδ T cell isolation, and gene expression in γδ T cells was evaluated before or after IFN-α treatment *in vitro*.

**Results:**

Although γδT cells decreased in CHB patients during pegIFN-α therapy, their capacities to produce TNF-α and to express CD107a were enhanced. More effector γδT cells (CD27-CD45RA+) were found in the response group than in non-response group. Furthermore, IFN-α boosted the expression of *Mx2* and cytokine genes in γδT cells from CHB patients *in vitro*.

**Conclusion:**

IFN-α could enhance the cytokine production or cytotoxicity potential of γδT cells *in vivo* and *in vitro*. The enhanced function of γδT cells might contribute to the effect of IFN-α treatment.

## Introduction

Chronic hepatitis B (CHB) is a serious public health problem in Eastern Asia. The disease is caused by persistent infection with hepatitis B virus (HBV), and has many complex clinical manifestations [1,2]. To date, interferon (IFN)-α is one of the main antiviral drugs against HBV, which has potential advantages compared to the nucleoside analogs including a lack of drug resistance, a finite and defined treatment course, and a higher likelihood of hepatitis B surface antigen (HBsAg) clearance. After about 10 years of pegylated-interferon (pegIFN)-α-based therapy for hepatitis B, pegIFN-α has attracted increased interest due to its more effective antiviral activity than standard IFN-α [3,4].

It is well established IFN-α induces anti-viral proteins and mediates the immune response. First, after interacting with the cell surface receptors, IFN-α activates the JAK-STAT signaling pathway. This results in transcription of interferon-stimulated genes (ISGs) and expression of proteins (protein kinase R, 2–5A oligosynthetase/RNAse L system, or the Mx system), which induce antiviral activity. Second, and perhaps more importantly, IFN-α directly or indirectly modulates the immune system including activation of nature killer (NK) cells, B cells, CD8^+^T cells, or production of some inflammatory cytokines[5,6].

In recent years, an increasing number of studies have shown that innate immune cells, especially NK cells, play an important role in the IFN-α treatment of viral hepatitis. Those studies have revealed that during IFN-α treatment, the specific T-cell response is suppressed but NK cells proliferate and are activated. Activation of NK cells in the early stage of IFN-α treatment is associated with the control of hepatitis C virus (HCV) infection [7,8].

γδ T cells, one type of innate immune cells, share many aspects of phenotype and function with NK cells. Similar to NK cells, γδ T cells express NKG2D, CD56 or other NK markers, exert strong cytotoxicity, and secrete different types of cytokines. As reported recently, γδ T cells have multifaceted immunological functions and might be a good immunotherapeutic tool for infectious diseases and tumors [9]. These cells display their activity either through directly affecting microbial growth, destroying the infected or transformed cells, or by modulating other immune functions, such as T helper (Th)1 immune response polarization, B cell activation, or dendritic cell maturation [10,11]. And Cimini’s study has shown that IFN-α improves phosphoantigen-induced Vγ9Vδ2 T cell production of IFN-γ during chronic HCV infection [12].

In our previous study, we found that the proportion of total γδ T cells increased in the blood of CHB patients, but the frequency of Vδ2 T cells decreased [13,14]. In this study, we further investigated the change in γδ T cells in CHB patients treated with IFN-α, and explored the possible effect of γδ T cells in the treatment of CHB with IFN-α.

## Materials and Methods

### Patients

Total fifteen treatment-naïve patients with CHB were selected from the Department of Infectious Diseases, Second Affiliated Hospital of Chongqing Medical University, China, during August 2011 to May 2013. All enrolled patients met the criteria for treatment with IFN-α, including serum alanine aminotransferase (ALT) level 80–400 U/L (2~10-fold the upper limit of normal), total bilirubin (TB) normal, HBV-DNA load 10^5^–10^8^ copies/mL, and positive HBsAg and hepatitis B e antigen (HBeAg) for at least six months. Patients were excluded if they were co-infected with other hepatitis viruses or had other liver diseases, such as hepatocellular carcinoma, autoimmune disease, or alcoholic hepatitis. Six healthy age-matched controls (HCs) were also recruited. In these 15 CHB patients, ten of them were recruited for the dynamic observation of peripheral γδ T cells during IFN-α treatment for 48 weeks. The other five patients before IFN-α treatment and six HCs were blood sampled for γδ T cell sorting and IFN-α treatment *in vitro*. The characteristics of all these subjects are shown in Tables 1 and 2.

**Table 1:** Clinical parameters of the 10 CHB patients enrolled for the dynamic observation of γδ T cells during pegIFN-α treatment for 48 weeks.

Sample	Sex	Age	At the beginning of treatment (week 0)				At the treatment endpoint (week 48)				Group
ID	(M/F)	(Year)	ALT	HBsAg	HBeAg	HBV-DNA	ALT	HBsAg	HBeAg	HBV-DNA	[Responder (R) or
			(IU/L)	(COI)	(COI)	(log_10_ copies/mL)	(IU/L)	(COI)	(COI)	(log_10_ copies/mL)	Nonresponder (N)]
1	M	26	104	6866	951.4	7.12	134	6584	2.11	6.09	N
2	M	38	107	793.7	1005	8.91	225	6637	0.59	7.42	N
3	M	28	395	1700	1075	8.81	295	3350	1.27	7.54	N
4	M	22	106	6098	185.5	7.81	33	6999	0.29	<3	R
5	M	28	129	6844	60.74	6.39	48	6530	0.31	<3	R
6	M	25	106	3469	1355	8.24	46	7230	690.9	5.63	N
7	M	26	143	5365	650.5	7.99	34	17.59	1.00	<3	R
8	M	28	211	2057	1114	7.88	32	1632	0.23	<3	R
9	M	28	123	2411	1076	8.65	43	545.3	64.7	<3	N
10	M	21	413	2179	784.9	8.22	42	4062	0.19	4.68	R

Abbreviations: CHB, chronic hepatitis B; pegIFN-α, pegylated-interferon α; ALT, alanine aminotransferase; HBsAg, hepatitis B surface antigen; HBeAg, hepatitis B e antigen; COI, cut-off index.

**Table 2:** The clinical parameters of 5 CHB patients with and 6 HCs enrolled for the peripheral γδ T cells isolation and IFN-α stimulation *in vitro* (Mean±SD).

Group	CHB	HC
**N**	5	6
**Sex(M/F)**	3/2	3/3
**Age(year)**	32.8±5.1	27.5±2.6
**ALT(IU/L)**	109.4±34.6	15.8±9.1
**AST(IU/L)**	73.4±20.5	22.3±5.3
**TB(μmol/L)**	14.5±3.3	11.6±2.3
**HBV-DNA(copies/mL)**	7.7±0.6	0

Abbreviations: CHB, chronic hepatitis B; HC, healthy control; ALT, alanine aminotransferase; AST, Aspertate Aminotransferase; TB, total bilirubin.

Peg-IFN-α -2a (Pegasys; Hoffman-La Roche, Shanghai, China) was administered subcutaneously at a dose of 180μg once weekly for 48 weeks. At the treatment endpoint, the patients who had normal ALT level, loss of HBeAg, and >3 log_10_ decrease in HBV-DNA were considered to be responders.

The trial was carried out with approval of the Ethics Committee of the Second Affiliated Hospital of Chongqing Medical University, and written informed consent was obtained from all participants.

### Clinical evaluation and HBV-DNA quantification

HBsAg and HBeAg were measured by the Roche electrochemiluminescence method. HBsAg and HBeAg values were evaluated by a cut-off index (COI): a COI of over 1.0 indicated a positive response. Hepatitis B surface antibody, hepatitis B e antibody and hepatitis B core antibody were detected with a commercial enzyme immunoassay kit. Serum HBV-DNA levels were determined by Roche real-time fluorescent quantitative polymerase chain reaction (PCR) (Lightcycler; Hoffman-La Roche,Swiss). The detection limit of this HBV-DNA assay was 1,000 copies/mL. All liver function parameters were evaluated using an automatic biochemical analyzer. The normal level of ALT, AST or TB was 6–48 IU/L, 10–45 IU/L, or 3–21 μmol/L.

### Detection of γδ T cell phenotype by fluorescence activated cell sorter (FACS)

Blood samples from 10 CHB patients were collected for determination of the phenotype and immune function of γδ T cells before and during IFN-α treatment at 4, 8, 12, 24, 36 and 48 weeks. The phenotypes T cell receptor (TCR) γδ, Vδ1, Vδ2, CD45RA or CD27 were detected according to the manufacturers’ instructions (BD Biosciences, La Jolla, CA, USA). The following fluorochrome-conjugated monoclonal antibodies (mAbs) were used: Peridinin chlorophyll (PerCP)-conjugated anti-CD3 mAb (clone SK7), allophycocyanin (APC)-conjugated anti-TCR γδ mAb (clone B1), phycoerythrin (PE)-conjugated anti-Vδ2 mAb (clone B6), PE-conjugated anti-CD45RA mAb, and fluorescein isothiocyanate (FITC)-conjugated anti-CD27 mAb, and were purchased from BD Biosciences(La Jolla, CA, USA). FITC-conjugated anti-Vδ1 mAb was purchased from Thermo Fisher Scientific (clone TS8.2; Rockford, IL, USA). The appropriate volume of antibody was added to 100 μL fresh peripheral anticoagulated blood and incubated for 30 minutes in the dark at 4°C. Erythrocytes were lysed using BD FACS lysing solution, and cells were washed in PBS supplemented with 1% fetal calf serum (FCS). Stained cells were immediately analyzed using the FACS Canto II flow cytometer (BD Immunocytometry Systems, San Jose, CA, USA). Data were analyzed using FACSDiva 2.0 software (BD Immunocytometry Systems).

### Intracellular cytokine staining (ICS)

At the same time as phenotype detection, expression of intracellular IFN-γ, tumor necrosis factor (TNF)-α, CD107a or Granzyme B was determined by ICS. Total leukocytes were obtained from whole blood by lysis of erythrocytes using PBS containing 0.85% NH_4_Cl. The cells were adjusted to ~5×10^6^/mL in RPMI 1640 culture medium supplemented with 10% FCS and stimulated with 100 ng/mL phorbol myristate acetate (PMA) plus 1 μg/mL ionomycin for 4 hours, in the presence of the secretion inhibitor monensin (BD Biosciences). Cells were stained with APC-conjugated anti-TCR γδ mAb, followed by washing with PBS, and fixation in 4% paraformaldehyde. Stained cells were permeabilized using 0.1% saponin (Sigma, St. Louis, MO, USA). Cells were incubated with FITC-conjugated anti-IFN-γ (clone 4S.B3; eBioscience, San Diego, CA, USA), PE-conjugated anti-TNF-α (clone Mab11; BD Biosciences), PE-conjugated anti-CD107a (clone H4A3; BD Biosciences), or FITC-conjugated anti-Granzyme B (clone GB11; BD Biosciences) for 30 minutes at 4°C. Finally, cells were washed with PBS, and analyzed using the FACS Canto II flow cytometer (BD Immunocytometry Systems). Data were analyzed using FACSDiva 2.0 software (BD Immunocytometry Systems).

### TCR γδ T cell isolation

Blood from five CHB patients before IFN-α treatment and six HCs (clinical characters showed in Table 2) was collected to generate γδ T cells from the buffy coat, and to determine the gene expression of γδ T cells when stimulated with IFN-α *in vitro*. Peripheral blood mononuclear cells (PBMCs) were obtained from 30 mL fresh peripheral anticoagulated blood by Ficoll separation (Ficoll-Paque), and TCR γδ T cells were isolated from the PBMCs by positive selection using the TCR γδ T cell isolation kit (Miltenyi Biotec, Bergisch Gladbach, Germany). The isolations were performed according to manufacturer’s instructions (Miltenyi Biotec, Bergisch Gladbach, Germany). Purity of the sorted TCR γδ T cells was determined by flow cytometry analysis. The results showed >95% purity of γδ T cells could be obtained in the acquired fractions.

### Analysis of mRNA expression of γδ T cells stimulated with IFN-α by quantitative RT-PCR

The sorted γδ T cells were divided into two parts: one for IFN-α treatment, and the other for the controls. Cells were cultured in RPMI 1640 medium supplemented with 10% FCS, 100 U/mL penicillin and 100μg/mL streptomycin in 5% CO_2_ at 37°C with or without recombinant human IFN-α-2a (1,000 U/mL) (Hoffman-La Roche, Shanghai, China) for 4 hours. Cellular RNA was isolated with Zymo mini RNA isolation kit (Zymo Research Corporation, Irvine, CA, USA). About 150 ng RNA was reverse transcribed with oligo(dT) primer using the PrimeScript RT Reagent Kit with gDNA Eraser (Perfect Real Time) (Takara Bio, Japan). Real-time PCR was performed using SYBR Green quantitative PCR by SYBR Premix Ex Taq II (Tli RnaseH Plus) (Takara Bio, Japan). Primer sequences are listed in Table 3. All the samples were detected in triplicate with 7300 PCR Detection System (Applied Biosystems, Foster City, CA, USA) for 40 cycles of 5 s at 95°C followed by 31 s at 60°C. GADPH was used as an internal control. Data were analyzed using the 2^-ΔΔCt^ method: gene expression was represented by Ct value, ΔCt = Ct(gene)-Ct(GAPDH), ΔΔCt = ΔCt(gene of HBV patients)-average ΔCt(gene of healthy controls), then one ΔΔCt would be used to produce the 2^-ΔΔCt^ value, which was displayed as a fold change in gene expression of γδ T cells from CHB patients, with or without IFN-α treatment versus healthy controls.

**Table 3:** The gene-specific primers for quantitative RT-PCR.

Gene	primers
***Ifit1* (NM_001548)**	forward: 5’-GCCTTGCTGAAGTGTGGAGGAA-3’
	reverse: 5’-ATCCAGGCGATAGGCAGAGATC-3’
***Isg15* (NM_005101)**	forward: 5’-CTCTGAGCATCCTGGTGAGGAA-3’
	reverse: 5’-AAGGTCAGCCAGAACAGGTCGT-3’
***Mx1* (NM_002462)**	forward: 5’-GGCTGTTTACCAGACTCCGACA-3’
	reverse: 5’-CACAAAGCCTGGCAGCTCTCTA-3’
***Mx2* (NM_002463)**	forward: 5’-AAAAGCAGCCCTGTGAGGCATG-3’
	reverse: 5’-GTGATCTCCAGGCTGATGAGCT-3’
***Cxcl*10 (NM_001565)**	forward: 5’-GGTGAGAAGAGATGTCTGAATCC-3’
	reverse: 5’-GTCCATCCTTGGAAGCACTGCA-3’
***Ifnγ*(NM_000619)**	forward: 5’-GAGTGTGGAGACCATCAAGGAAG-3’
	reverse: 5’-TGCTTTGCGTTGGACATTCAAGTC-3’
***Tnfα*(NM_000594)**	forward: 5’-CTCTTCTGCCTGCTGCACTTTG-3’
	reverse: 5’-ATGGGCTACAGGCTTGTCACTC-3’
***Il10* (NM_000572)**	forward: 5’-TCTCCGAGATGCCTTCAGCAGA-3’
	reverse: 5’-TCAGACAAGGCTTGGCAACCCA-3’
***Il12a* (NM_000882)**	forward: 5’-TGCCTTCACCACTCCCAAAACC-3’
	reverse: 5’-CAATCTCTTCAGAAGTGCAAGGG-3’

### Statistical analysis

All data were analyzed by SAS version 9.2 (SAS Institute, Cary, NC, USA). Repeated measures analysis of variance and simple-effects analysis were used to compare the change in proportion of total γδ T cells or the certain subtype of γδ T cells from responders and non-responders, or from the different treatment time points. A two-sided p value <0.05 was considered as significant. For the comparison of gene expression *in vitro*, the gene expression from HCs was set as “1”, and the significant change in gene expression from CHB patients was set as at least two fold (upregulation) or 0.5-fold (downregulation).

## Results

### The proportion of γδ T cells in CHB patients decreased during pegIFN-α therapy

**Fig 1:**
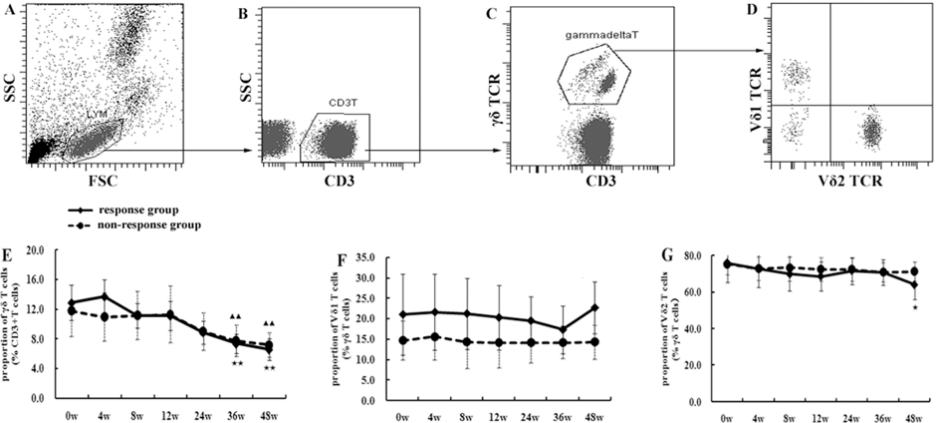
The changes in the proportion of total γδ T cells or the subtype of Vδ1 T or Vδ2 T cells from 10 CHB patients during pegIFN-α treatment. The FACS figures were the representatives of gated lymphocytes (A), CD3 T cells (B), γδ T cells (C), or Vδ1 T or Vδ2 T cells (D). The time curves showed the proportion of γδ T cells (E), Vδ1 T (F) or Vδ2 T (G) cells during the treatment for 48 weeks. The statistical analyses were performed between the response (5 patients) and non-response group (5 patients), and also among different time points in each group. When comparing a certain timepoint to others, there showed ★ (p<0.05) or ★★ (p<0.01) in response group and ▲ (p<0.05) or ▲▲ (p<0.01) in non-response group.

FACS gating and Figures of γδ T, Vδ1 or Vδ2 T cells were shown in Fig. 1A-D. The proportions of γδ T cells from responders or non-responders all declined consistently during treatment, from 12.9% or 11.8% before treatment to 6.6% or 7.2% at week 48 in treatment (Fig. 1E). These differences between responders and non-responders at each time point were not significant (p>0.05), but the proportion of γδ T cells at week 36 or 48 was significantly lower than that at week 0, 4, 8 and 12 (p<0.01). The proportion of Vδ1T cells from responders was higher than in non-responders at every time point, but no significant differences were shown between the two groups (p>0.05). The proportion of Vδ1 T cells from responders was 21.1% at baseline, then it decreased slightly to 17.3% at week 36, but it increased to 22.7% at week 48. (Fig. 1F). There was no significant difference in the proportion of Vδ1 T cells between each time point in responders or non-responders (p>0.05). The proportion of Vδ2 T cells from responders decreased sharply from 75.8% before treatment to 64.0% at the end of treatment, (Fig. 1G) and the difference between week 48 and week 0, 4, 24 or 36 was significant (p<0.05). However, the differences between the responders and non-responders were not significant (p>0.05).

### Responders had higher percentage of effector γδ T cells than non-responders during pegIFN-α treatment

**Fig 2:**
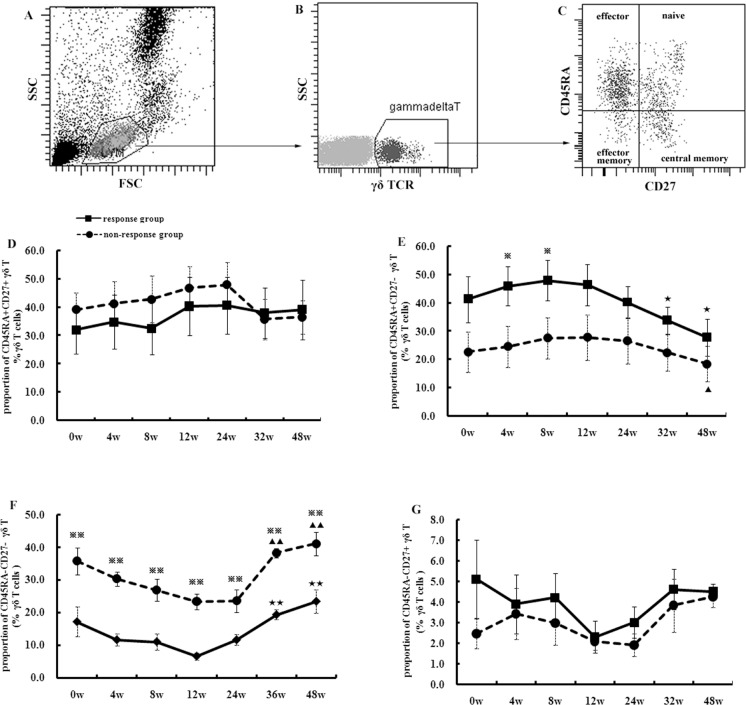
The dynamic change in naïve or effector γδ T cells from 10 CHB patients during pegIFN-α therapy. FACS gating strategy was shown in A, B and C. The time curves showed the proportion of CD45RA+CD27+ γδ T cells (naïve) (D), CD45RA+CD27- γδ T cells (effector) (E), CD45RA-CD27- γδ T cells (effector memory) (F), or CD45RA-CD27+ γδ T cells (central memory) (G) during the treatment for 48 weeks. The statistical analyses were performed between the response (5 patients) and non-response group (5 patients), and also among different time points in each group. When comparing a certain timepoint to others, there showed ★ (p<0.05) or ★★ (p<0.01) in response group and ▲ (p<0.05) or ▲▲ (p<0.01) in non-response group. When comparing response to non-response group, there showed ※ (p<0.05) or ※※ (p<0.01) at a certain timepoint.

CD45RA and CD27 molecules were used to investigate the effector or memory phenotype of γδ T cells. The γδ T cells could be divided as the naïve cells (CD45RA^+^CD27^+^), effector cells (CD45RA^+^CD27^-^), effector memory cells (CD45RA^-^CD27^-^), or central memory cells (CD45RA^-^CD27^+^) (FACS Fig. 2A-C). The proportion of naïve γδ T cells increased from 32.1% to 40.7% in the responders and 39.2% to 48.1% in the non-responders during 24 weeks treatment, and then declined to 39.1% in the responders and 36.6% in the non-responders at week 48 (Fig. 2D). Although the proportion of naïve γδ T cells in the non-responders was higher than in the responders, no significant difference was found between the two groups or among the time points (p>0.05). The percentage of effector γδ T cells at the outset of treatment was 22.6% and 41.3% for non-responders and responders, respectively, and increased to 27.9% and 47.9% after 8 weeks, and finally dropped to 18.4% and 27.8% (Fig. 2E). The frequency of effector γδ T cells from responders was significantly higher than from non-responders at week 4 and 8 in treatment (p<0.05). The non-responders patients showed a significantly higher proportion of effector memory γδ T cells (Fig. 2F) than the responders at each time point from week 0 (35.7% vs. 17.1%), week 12 (23.3% vs. 6.6%), to week 48 (41.0% vs. 23.4%) (p<0.01). There were few central memory γδ T cells detected in the peripheral blood and the percentage fluctuated during treatment (Fig. 2G), and no significant differences were shown between the two groups or among the time points (p>0.05).

### Increased percentage of TNF-α- and CD107a-producing γδ T cells were found in CHB patients during pegIFN-α treatment

**Fig 3:**
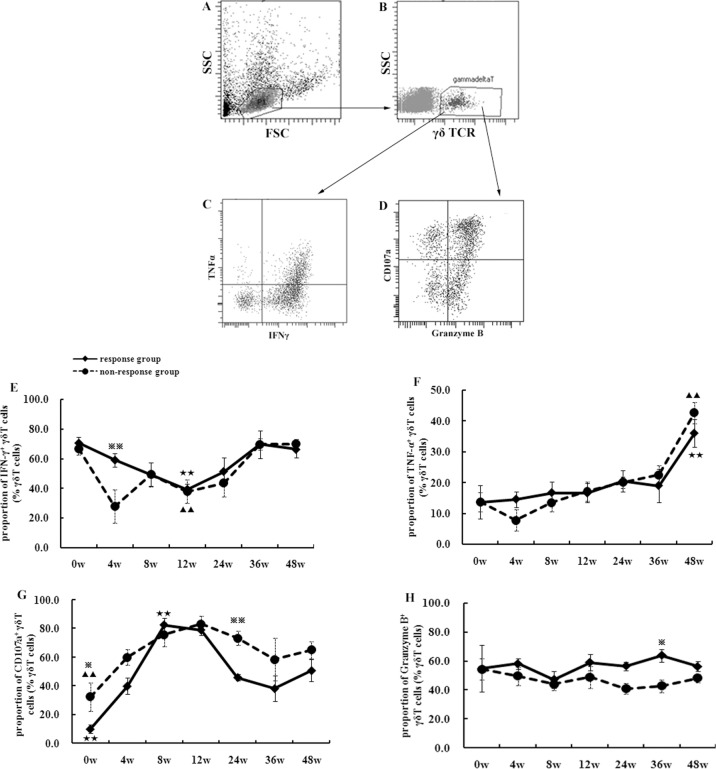
The changes in function of γδ T cells from 10 CHB patients during pegIFN-α therapy. FACS figures showed IFNγ+, TNFα+, CD107a+ or Granzyme B+ γδ T cells (A-D). The curves showed dynamic changes in proportion of IFNγ+ γδ T cells (E), TNFα+ γδ T cells (F), CD107a+ γδ T cells (G), or Granzyme B+ γδ T cells (H) during the pegIFN-α therapy for 48 weeks. The statistical analyses were performed between the response (5 patients) and non-response group (5 patients), and also among different time points in each group. When comparing a certain timepoint to others, there showed ★ (p<0.05) or ★★ (p<0.01) in response group and ▲ (p<0.05) or ▲▲ (p<0.01) in non-response group. When comparing response to non-response group, there showed ※ (p<0.05) or ※※ (p<0.01) at a certain timepoint.

The most important properties of functional γδ T cells include cytokine production and cytotoxicity. Fig. 3A-D shows IFN-γ-, TNF-α-, Granzyme-B- and CD107a- producing γδ T cells, respectively. In the responders, the percentage of IFN-γ^+^γδ T cells declined from 70.5% at baseline to 39.2% at week 12, then increased to 66.3% at week 48 (Fig. 3E). There was a sharp decrease in the frequency of IFN-γ^+^γδ T cells in non-responders from 66.8% at 0 weeks to 27.8% at week 4, followed by an increase to 70.0% at the end of treatment. A significant difference in the percentage of IFN-γ^+^γδ T cells at week 4 was shown between responders and non-responders (p<0.01). For each group, there was a significant difference between week 12 and the other times (p<0.01). The percentage of TNF-α^+^γδ T cells increased from 13.6% or 13.7% at week 0 to 36.0% or 42.6% at week 48 for responders or non-responders, respectively (Fig. 3F). There were no significant differences between these two groups at each point (p>0.05), but the frequency at 48 weeks was significantly higher than at other time points for each group (p<0.01).

The percentage of Granzyme-B^+^γδ T cells from the responders peaked as 63.7% at week 36 (Fig. 3H), and it was significantly higher than that from non-responders (p<0.05). However, no significant differences were found between each time point in each group (p>0.05). As shown in Fig. 3G, the frequency of CD107a^+^γδ T cells increased markedly from 10.1% or 32.5% at week 0 to 82.4% at week 8 or 83.3% at week 12 in responders or non-responders, respectively. The frequency at week 0 or 24 from the non-responders was significantly higher than that from the responders (p<0.05).

### *Mx2* expression in IFN-α-treated γδ T cells from CHB patients showed significantly higher than that from HC

**Fig 4:**
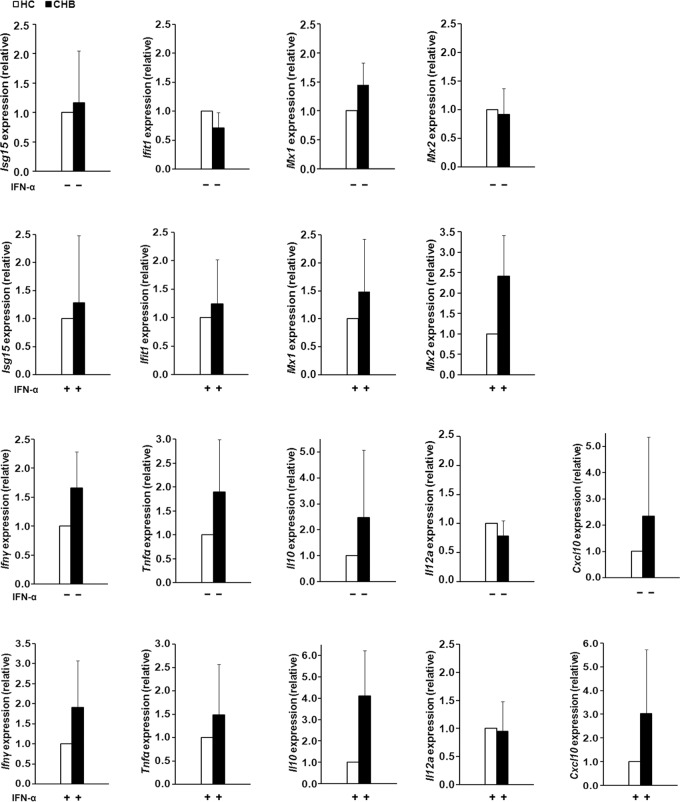
Gene expression of γδ T cells with (B, D) or without (A, C) IFN-α stimulation *in vitro*. Peripheral γδ T cells were isolated from 6 healthy controls (HC) or 5 patients with chronic HBV infection (CHB). The expression of *Isg15*, *Ifit1*, *Mx1* and *Mx2* was shown in A and B, and expression of *Ifnγ*, *Tnfα*, *Il10*, *Il12a*, or *Cxcl10* was shown in C and D. All genes expression from peripheral γδ T cells was normalized to that of GAPDH and results of 5 CHB patients are presented with fold (Mean±SD) relative to the average of 6 HCs. The difference in the gene expression was significant when it was higher than 2-fold or lower than 0.5-fold.

We investigated changes in gene expression when γδT cells were directly treated with IFN-α. Some IFN-α-induced genes, *Ifit1*, *Isg15*, *Mx1* and *Mx2*, were detected (Fig. 4A-B). Among these genes, *Isg15* and *Mx1* expression in untreated-γδT cells from CHB patients was higher (but not significantly) than that from HCs. When compared to IFN-α-untreated γδT cells, expression of these four genes increased in the IFN-α-stimulated cells from HCs or CHB patients (data not shown). When compared to IFN-α-stimulated γδT cells from HCs, *Mx2* gene expression from CHB patients showed a significant increase at 2.4-fold higher than that from HCs.

### Elevated mRNA expression of *Ifnγ*, *Tnfα*, *Il10*, *Il12* and *Cxcl10* gene in γδ T cells from CHB patients after IFN-α treatment

The expression of some cytokine or chemokine genes in γδ T cells was detected before and after IFN-α treatment (Fig. 4C-D). Before IFN-α stimulation, expression of *Ifnγ*, *Tnfα*, *Il10* and *Cxcl10* from CHB patients increased, but *Il12a* decreased compared to that of healthy controls. IFN-α enhanced expression of these genes in HCs and CHB patients, with a 2~4-fold increase in IFN-α-treated γδ T cells for *Ifng*, *Tnfa*, *Il10* and *Il12* expression, and about 300-fold increase for *Cxcl10* expression. In comparison with IFN-α-stimulated γδT cells from HCs, *Il10* and *Cxcl10* gene expression from CHB patients displayed a significant increase compared with that from HCs.

## Discussion

CHB is a serious disease, especially in Eastern Asian countries. The immune disorder in these patients is characterized as specific immunological inadequacy to HBV and nonspecific hyperimmune reaction to hepatocytes. As a result, persistent HBV infection can be accompanied by persistent liver inflammation [15,16]. To date, IFN-α is one of the most important anti-HBV treatments, whose mechanism includes a direct influence on HBV RNA, capsid, or covalently closed circular DNA stability in hepatocytes, and more importantly, it can exert an immunoregulatory function to improve CD4^+^ and CD8^+^ T cells, NK cells or other immune cells [6–8]. However, few studies have demonstrated the changes in γδT cells during IFN-α treatment.

γδT cells are one type of innate immune cells, and their functions include cytotoxicity, cytokine secretion, antigen presentation, and crosstalk with other immune cells. In the process of infection, autoimmune reaction, or tumor development, γδT cells are activated to stimulate, regulate, or even suppress the immune response [17–19]. γδT cells are mainly distributed in the epithelial tissues, such as the gut, skin, lung, or liver. The percentage of total γδT cells in peripheral blood is <10% of T cells. And the major (>70%) subtype of peripheral γδT cells is Vδ2T cells. However, in skin, gut or some other epithelial tissues, the subtype of Vδ1T cells constitutes the major population.

In the present study, dynamic changes in the percentage of γδT, Vδ1T or Vδ2T cells were detected. The percentage of total γδT cells decreased during treatment with IFN- in responders and non-responders. It was particularly interesting to find that all the percentages of Vδ1T cells during treatment in the responders were higher than in the non-responders. In our previous study, increased percentages of circulating Vδ1T cells and decreased Vδ2T cells were found in patients with CHB [13]. These data imply some important role of Vδ1T in anti-HBV immunity. Vδ1T cells were reported to recognize lipids presented by CD1c/d, MICA/B, or some stress-induced self-antigens. This differs from Vδ2T cells that uniquely respond to non-peptide alkylphosphates [20,21]. Expansion of Vδ1T cells has been shown in infection with HIV-1 and cytomegalovirus, and expansion of Vδ2T cells occurs in infection with mycobacterial species, *Listeria monocytogenes*, and Epstein-Barr virus. Certain antigens expressed on tissue cells might result in expansion of different subtypes of γδT cells [22,23].

According to the surface expression of CD45RA and CD27, human γδT cells can be divided into four subsets: naïve (CD45RA^+^CD27^+^), central memory (CD45RA^-^CD27^+^), effector memory (CD45RA^-^CD27^-^), and effector cells (CD45RA^+^CD27^-^) [24–27]. In our study, the circulating γδT cells were largely composed of naïve, effector memory, and effector cells. Few central memory γδT cells could be detected in the circulating γδT cells. Our results also showed a higher percentage of late-stage effector (CD45RA^+^CD27^-^) γδT cells in the responders than non-responders. These results demonstrated that the γδT cells in the responders might have greater potential to respond to antigen and expand to the end stage of effector cells.

IFN-γ and TNF-α were the main cytokines produced in the activated γδT cells [28, 29]. We found that the production of IFN-γ decreased during the first 12 weeks of treatment and then it gradually increased, and there was an increasing trend in TNF-α production throughout the treatment course. IFN-α could enhance RNA expression of IFN-γ gene in γδT cells *in vitro*. The results revealed that interaction of γδT cells with other immune cells could influence IFN-γ production. It also implies that γδT cells are heterogeneous and different types of γδT cells have different patterns of cytokine production. So, the changing trend of IFN-γ production was not in accordance with that of TNF-α production.

CD107a (lysosomal-associated membrane protein-1) has been described as a functional marker of killer cell activity on CD8^+^ T, NK, γδT, or other immune cells [30]. During the first 12 weeks of IFN-α treatment, the percentage of CD107a^+^ γδT cells increased dramatically from 10.1% (average) to 82.4% in the responders and 32.5% to 83.3% in the non-responders. Then, it decreased to 50.7% in the responders and 65.1% in the non-responders by the end of IFN-α treatment. Granzyme B is a caspase-like serine protease commonly found in the granules of cytotoxic lymphocytes or NK cells [31]. In our study, almost half of γδT cells could express Granzyme B from the beginning to the end of the treatment. Our results demonstrated that CD107a expression might be a sensitive marker for the cytotoxic activity of γδT cells when stimulated with IFN-α.

In addition, to observing the direct effect of IFN-α on γδT, the γδT cells were isolated from CHB patients and HCs for *ex vivo* experiments. Our results show IFN-α increased expression of all the observed genes. *Isg15*, *Ifit1*, *Mx1* and *Mx2* are among the best defined ISGs in immune cells [32]. We demonstrated that after IFN-α treatment, expression of *Ifit1* and *Mx2* in CHB patients increased very much and became higher than that in HCs, especially the significant increase for *Mx2*. Although MX1 and MX2 are closely related proteins, they exert different functions in different viral infections. Goujon *et al*. have shown that MX2 suppresses HIV-1 infection, but MX1 does not. In contrast, MX1 is an inhibitor of influenza A virus, but MX2 is ineffective [33]. For HBV infection, MX1 protein inhibits viral replication by interaction with hepatitis B core antigen [34]. Our results showed that the different pattern of *Mx1* and *Mx2* RNA expression in γδT cells from CHB patients before or after IFN-α stimulation.

γδT cells have a complicated cytokine profile. When activated, they secrete Th1 type cytokines (IFN-γ and TNF-α), or Th2 type cytokines [interleukin (IL)-4 and IL-10], and they produce IL-12 or other cytokines similar to antigen presenting cells. Also, chemokines are produced in γδT cells [35]. Our results showed that before IFN-α stimulation, expression of *Ifng*, *Tnfa* and *Il10* increased, but the expression of *Il12a* and *Cxcl10* decreased. Once stimulated with IFN-α, the Th1 and Th2 cytokines were all enhanced in CHB patients. These results suggested that the γδT cells from CHB patients were heterogeneous and had different functions. This study also showed that during PegIFN-α therapy, the function of γδ T cells was enhanced, and these cells might transfer into liver tissue and exert their effects. So it appeared that decreased percentage of γδ T cells and increased function could exist simultaneously.

In conclusion, we demonstrated that, although the proportion of total γδT cells decreased during IFN-α treatment, their functions of TNF-α production and CD107a expression were enhanced. The responders had more effector γδT cells than the non-responders had. *In vitro*, IFN-α boosted many ISGs in γδT cells. However, it was not the same *in vivo*, due to the complicated factors affecting the function of γδT cells.

## References

[1] Liang TJ. Hepatitis B: the virus and disease. Hepatology. 2009; 49: S13–21. 10.1002/hep.22881 19399811 PMC2809016

[2] Liaw YF, Chu CM. Hepatitis B virus infection. Lancet. 2009; 373: 582–592. 10.1016/S0140-6736(09)60207-5 19217993

[3] Parmar S, Platanias LC. Interferons: mechanisms of action and clinical applications. Curr Opin Oncol. 2003;15: 431–439. 14624225 10.1097/00001622-200311000-00005

[4] Craxi A, Cooksley WG. Pegylated interferons for chronic hepatitis B. Antiviral Res. 2003; 60: 87–89. 14638403 10.1016/j.antiviral.2003.08.015

[5] Belloni L, Allweiss L, Guerrieri F, Pediconi N, Volz T, Pollicino T, et al IFN-α inhibits HBV transcription and replication in cell culture and in humanized mice by targeting the epigenetic regulation of the nuclear cccDNA minichromosome. J Clin Invest. 2012; 122: 529–537. 10.1172/JCI58847 22251702 PMC3266786

[6] Dill MT, Makowska Z, Trincucci G, Gruber AJ, Vogt JE, Filipowicz M, et al Pegylated IFN-α regulates hepatic gene expression through transient Jak/STAT activation. J Clin Invest. 2014; 124: 1568–1581. 10.1172/JCI70408 24569457 PMC3973080

[7] Ahlenstiel G, Edlich B, Hogdal LJ, Rotman Y, Noureddin M, Feld JJ, et al Early Changes in Natural Killer Cell Function Indicate Virologic Response to Interferon Therapy for Hepatitis C. Gastroenterology. 2011; 141: 1231–1239. 10.1053/j.gastro.2011.06.069 21741920 PMC3353552

[8] Micco L, Peppa D, Loggi E, Schurich A, Jefferson L, Cursaro C, et al Differential boosting of innate and adaptive antiviral responses during pegylated-interferon-alpha therapy of chronic hepatitis B. J Hepatol. 2013; 58: 225–233. 10.1016/j.jhep.2012.09.029 23046671

[9] Lopez RD. Human gammadelta-T cells in adoptive immunotherapy of malignant and infectious diseases. Immunol Res. 2002; 26: 207–221. 12403359 10.1385/IR:26:1-3:207

[10] Bonneville M, O'Brien RL, Born WK. Gammadelta T cell effector functions: a blend of innate programming and acquired plasticity. Nat Rev Immunol. 2010; 10: 467–478. 10.1038/nri2781 20539306

[11] Vantourout P, Hayday A. Six-of-the-best: unique contributions of γδT cells to immunology. Nat Rev Immunol. 2013; 13: 88–100. 10.1038/nri3384 23348415 PMC3951794

[12] Cimini E, Bonnafous C, Bordoni V, Lalle E, Sicard H, Sacchi A, et al Interferon-α improves phosphoantigen-induced Vγ9Vδ2 T-cells interferon-α production during chronic HCV infection. PLoS One. 2012; 7: e37014 10.1371/journal.pone.0037014 22629350 PMC3358305

[13] Chen M, Zhang D, Zhen W, Shi Q, Liu Y, Ling N, et al Characteristics of circulating T cell receptor gamma-delta T cells from individuals chronically infected with hepatitis B virus (HBV): an association between V(delta)2 subtype and chronic HBV infection. J Infect Dis. 2008; 198: 1643–1650. 10.1086/593065 18954265

[14] Chen M, Hu P, Peng H, Zeng W, Shi X, Lei Y, et al Enhanced Peripheral γδT Cells Cytotoxicity Potential in Patients with HBV-Associated Acute-On-Chronic Liver Failure Might Contribute to the Disease Progression. J Clin Immunol. 2012; 32: 877–885. 10.1007/s10875-012-9678-z 22415432

[15] Tan AT, Koh S, Goh V, Bertoletti A. Understanding the immunopathogenesis of chronic hepatitis B virus: an Asian prospective. J Gastroenterol Hepatol. 2008; 23: 833–843. 10.1111/j.1440-1746.2008.05385.x 18565018

[16] Zhang Z, Zhang S, Zou Z, Shi J, Zhao J, Fan R, et al Hypercytolytic activity of hepatic natural killer cells correlates with liver injury in chronic hepatitis B patients. Hepatology. 2011; 53: 73–85. 10.1002/hep.23977 21254163 PMC3767982

[17] Ajuebor MN, Jin Y, Gremillion GL, Strieter RM, Chen Q, Adegboyega PA. GammadeltaT cells initiate acute inflammation and injury in adenovirus-infected liver via cytokine-chemokine cross talk. J Virol. 2008; 82: 9564–9576. 10.1128/JVI.00927-08 18667515 PMC2546965

[18] Barcy S, De Rosa SC, Vieira J, Diem K, Ikoma M, Casper C, et al Gamma delta+ T cells involvement in viral immune control of chronic human herpesvirus 8 infection. J Immunol. 2008; 180: 3417–3425. 18292568 10.4049/jimmunol.180.5.3417

[19] Pinheiro MB, Antonelli LR, Sathler-Avelar R, Vitelli-Avelar DM, Spindola-de-Miranda S, Guimarães TM, et al CD4-CD8-αβ and γδ T cells display inflammatory and regulatory potentials during human tuberculosis. PLoS One. 2012; 7: e50923 10.1371/journal.pone.0050923 23239994 PMC3519797

[20] Siegers GM, Lamb LS Jr. Cytotoxic and Regulatory Properties of Circulating Vδ1+ γδT Cells: A New Player on the Cell Therapy Field? Mol Ther. 2014; 22: 1416–1422. 10.1038/mt.2014.104 24895997 PMC4435582

[21] Luoma AM, Castro CD, Mayassi T, Bembinster LA, Bai L, Picard D, et al Crystal structure of Vδ1 T cell receptor in complex with CD1d-sulfatide shows MHC-like recognition of a self-lipid by human γδ T cells. Immunity. 2013; 39: 1032–1042. 10.1016/j.immuni.2013.11.001 24239091 PMC3875342

[22] Girardi M. Immunosurveillance and immunoregulation by gammadelta T cells. J Invest Dermatol. 2006; 126: 25–31. 16417214 10.1038/sj.jid.5700003

[23] Chen CY, Yao S, Huang D, Wei H, Sicard H, Zeng G, et al Phosphoantigen/IL2 expansion and differentiation of Vγ2Vδ2 T cells increase resistance to tuberculosis in nonhuman primates. PLoS Pathog. 2013; 9: e1003501 10.1371/journal.ppat.1003501 23966854 PMC3744401

[24] Qin G, Liu Y, Zheng J, Xiang Z, Ng IH, Malik Peiris JS, et al Phenotypic and functional characterization of human γδT-cell subsets in response to influenza A viruses.J Infect Dis. 2012; 205: 1646–1653. 10.1093/infdis/jis253 22457284

[25] Di Mitri D, Azevedo RI, Henson SM, Libri V, Riddell NE, Macaulay R, et al Reversible senescence in human CD4+CD45RA+CD27- memory T cells. J Immunol. 2011; 187: 2093–2100. 10.4049/jimmunol.1100978 21788446

[26] Gioia C, Agrati C, Casetti R, Cairo C, Borsellino G, Battistini L, et al Lack of CD27-CD45RA-V gamma 9V delta 2+ T cell effectors in immunocompromised hosts and during active pulmonary tuberculosis. J Immunol. 2002; 168: 1484–1489. 11801693 10.4049/jimmunol.168.3.1484

[27] Angelini DF, Borsellino G, Poupot M, Diamantini A, Poupot R, Bernardi G, et al FcgammaRIII discriminates between 2 subsets of Vgamma9Vdelta2 effector cells with different responses and activation pathways. Blood. 2004; 104: 1801–1807. 15178578 10.1182/blood-2004-01-0331

[28] Christmas SE, Meager A. Production of interferon-gamma and tumour necrosis factor-alpha by human T-cell clones expressing different forms of the gamma delta receptor. Immunology. 1990; 71: 486–492. 2126252 PMC1384867

[29] Brinkmann V, Geiger T, Alkan S, Heusser CH. Interferon alpha increases the frequency of interferon gamma-producing human CD4+ T cells. J Exp Med. 1993; 178: 1655–1663. 8228812 10.1084/jem.178.5.1655 PMC2191249

[30] Alter G1, Malenfant JM, Altfeld M. CD107a as a functional marker for the identification of natural killer cell activity. J Immunol Methods. 2004; 294: 15–22. 15604012 10.1016/j.jim.2004.08.008

[31] Trapani JA, Sutton VR. Granzyme B: pro-apoptotic, antiviral and antitumor functions. Curr Opin Immunol. 2003; 15: 533–543. 14499262 10.1016/s0952-7915(03)00107-9

[32] de Veer MJ, Holko M, Frevel M, Walker E, Der S, Paranjape JM, et al Functional classification of interferon-stimulated genes identified using microarrays. J Leukoc Biol. 2001; 69: 912–920. 11404376

[33] Goujon C, Moncorgé O, Bauby H, Doyle T, Ward CC, Schaller T, et al Human MX2 is an interferon-induced post-entry inhibitor of HIV-1 infection. Nature. 2013; 502: 559–562. 10.1038/nature12542 24048477 PMC3808269

[34] Li N, Zhang L, Chen L, Feng W, Xu Y, Chen F, et al MxA inhibits hepatitis B virus replication by interaction with hepatitis B core antigen. Hepatology. 2012; 56: 803–811. 10.1002/hep.25608 22271421

[35] Carding SR, Egan PJ. Gammadelta T cells: functional plasticity and heterogeneity. Nat Rev Immunol. 2002; 2: 336–345. 12033739 10.1038/nri797

